# Convergent and Divergent Mechanisms of Epileptogenesis in mTORopathies

**DOI:** 10.3389/fnana.2021.664695

**Published:** 2021-04-09

**Authors:** Lena H. Nguyen, Angélique Bordey

**Affiliations:** ^1^Department of Neurosurgery, Yale School of Medicine, Yale University, New Haven, CT, United States; ^2^Department of Cellular & Molecular Physiology, Yale School of Medicine, Yale University, New Haven, CT, United States

**Keywords:** neuron migration, tuberous sclerosis complex, focal cortical dysplasia, GATOR1 complex, *in utero* electroporation, mTOR, cortical development, epilepsy

## Abstract

Hyperactivation of the mechanistic target of rapamycin complex 1 (mTORC1) due to mutations in genes along the PI3K-mTOR pathway and the GATOR1 complex causes a spectrum of neurodevelopmental disorders (termed mTORopathies) associated with malformation of cortical development and intractable epilepsy. Despite these gene variants’ converging impact on mTORC1 activity, emerging findings suggest that these variants contribute to epilepsy through both mTORC1-dependent and -independent mechanisms. Here, we review the literature on *in utero* electroporation-based animal models of mTORopathies, which recapitulate the brain mosaic pattern of mTORC1 hyperactivity, and compare the effects of distinct PI3K-mTOR pathway and GATOR1 complex gene variants on cortical development and epilepsy. We report the outcomes on cortical pyramidal neuronal placement, morphology, and electrophysiological phenotypes, and discuss some of the converging and diverging mechanisms responsible for these alterations and their contribution to epileptogenesis. We also discuss potential therapeutic strategies for epilepsy, beyond mTORC1 inhibition with rapamycin or everolimus, that could offer personalized medicine based on the gene variant.

## Introduction

mTORopathies comprise several neurodevelopmental disorders, including tuberous sclerosis complex (TSC), focal cortical dysplasia type II (FCDII), hemimegalencephaly (HME), and polyhydramnios, megalencephaly, and symptomatic epilepsy (PMSE) syndrome, among others. These disorders are characterized by hyperactivation of the mechanistic target of rapamycin (mTOR) signaling, malformation of cortical development (MCD), and intractable epilepsy (Crino, 2015). Recent advances in genomics have identified genetic mutations in regulators of the mTOR pathway as a common molecular etiology for TSC, FCDII, HME, and PMSE, thus emphasizing a central role for mTOR in the pathogenesis of these disorders (Marsan and Baulac, 2018; Muhlebner et al., 2019). The mTOR signaling pathway regulates cell growth and metabolism via two protein complexes, mTORC1 and mTORC2. Here, we focus on mTORC1 because all the identified pathogenic gene variants thus far impinge on mTORC1 activity, and information on mTORC2 activity is limited for these neurodevelopmental disorders.

**FIGURE 1:**
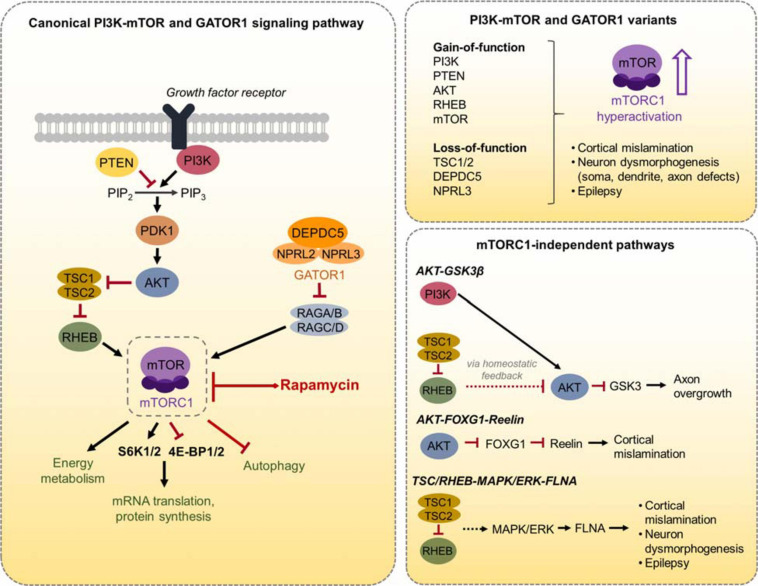
Schematic diagram of the canonical PI3K-mTOR and GATOR1 signaling pathway and mTORC1-independent pathways. **(Left)** The canonical PI3K-mTOR signaling pathway, the GATOR1 complex, and several mTORC1 downstream effectors are shown. mTORC1 regulates mRNA translation and protein synthesis via direct phosphorylation of S6K1/2 and 4E-BP1/2. mTORC1 also regulates energy metabolism and autophagy, as well as other processes not shown here. **(Right, top)** A summary of the PI3K-mTOR and GATOR1 gene variants discussed in this review is depicted. These variants converge on mTORC1 hyperactivation, leading to cortical mislamination, neuron dysmorphogenesis, and epilepsy. **(Right, bottom)** The three mTORC1-independent pathways that are discussed in this review and the associated phenotypes are depicted.

The canonical mTORC1 signaling pathway consists of phosphoinositide 3-kinase (PI3K), the activity of which is negatively regulated by phosphatase and tensin homolog (PTEN), AKT, tuberous sclerosis complex 1/2 (TSC1/2) protein complex, Ras homolog enriched in brain (RHEB), and mTORC1 (Dibble and Cantley, 2015) (Figure 1). mTORC1 signaling in response to cellular nutrients is additionally regulated by the GAP activity toward Rags 1 (GATOR1) protein complex (Bar-Peled and Sabatini, 2014). To date, numerous pathogenic gene variants in the PI3K-mTOR pathway and GATOR1 complex have been identified as genetic causes leading to mTORC1 hyperactivity, MCD, and epilepsy, and more will undoubtfully emerge with increasing accessibility to advanced genomic tools (Marsan and Baulac, 2018; Muhlebner et al., 2019). The role of mTOR signaling pathway in the brain has been the subject of many reviews (e.g., Lasarge and Danzer, 2014; Lipton and Sahin, 2014; Takei and Nawa, 2014; Switon et al., 2017) and is therefore not extensively discussed here. Rather, the aim of this review is to examine neuronal molecular and functional alterations resulting from distinct PI3K-mTOR and GATOR1 gene variants that converge on mTORC1 activation and discuss the potential mechanisms leading to epileptogenesis due to these pathological variants.

We first provide an overview of the distinct PI3K-mTOR or GATOR1 gene variants and introduce different animal models aimed to recapitulate the disease phenotypes. In particular, we discuss the advantages of *in utero electroporation* (IUE)-based models to replicate the genetic mosaicism as well as the focal nature of MCD observed in TSC and FCDII. We then review the current literature on IUE-based animal models of TSC/FCDII targeting cortical pyramidal neurons, which at this time comprises 33 studies summarized in Table 1. We compare the outcomes of distinct PI3K-mTOR and GATOR1 gene variants on the structural alterations that are pathological hallmarks of TSC/FCDII, namely cell misplacement and neuronal hypertrophy. We discuss these findings in the context of major molecular processes downstream mTORC1 that have known contributions to these specific alterations, such as cap-dependent translation. We next compare the electrophysiological properties and synaptic activities of neurons expressing the distinct gene variants. We focus on cell autonomous mechanisms of neuronal hyperexcitability, although non-cell autonomous changes in the surrounding environment such as decreased interneuron density (Zhong et al., 2021), altered connectivity of non-targeted, contralateral neurons (Onori et al., 2020), and increased vascular density (Zhang et al., 2019) have been reported in these models. One major finding we discuss is the abnormal expression of an mTORC1-dependent hyperpolarization-activated cyclic nucleotide-gated (HCN) channel isoform 4, HCN4, which provides a cyclic AMP (cAMP)-dependent mode of hyperexcitability in mutant pyramidal neurons expressing constitutively active *Rheb* (Hsieh et al., 2016). However, it remains uncertain whether these channels are present in neurons expressing the other gene variants. Both structural and functional changes will be discussed in a broader context for how they contribute to epileptogenesis. We then diverge from an mTOR-centric view by highlighting specific parallel molecular players and pathways that are additionally altered as a consequence of some, but not all, gene variants. We particularly examine the activities of AKT-Glycogen synthase kinase 3 beta (GSK3β) on axon growth, AKT-Forkhead box G1 (FOXG1)-reelin on neuronal migration, and TSC/RHEB-mitogen-activated protein kinase (MAPK)/ERK-filamin A (FLNA) on neuronal dysmorphogenesis and seizures. Finally, we discuss how these novel findings can lead to new treatment options in addition to using classical mTOR inhibitors such as rapamycin and everolimus.

**TABLE 1:** Summary of IUE-based rodent models of mTORopathies.

Gene	IUE age, cortical area	Migration defect/ misplacement (M), cytomegaly (C), Dendrite overgrowth (D)	Synaptic function and electrophysiological properties	Seizure phenotype	Other phenotypes	Pharmacological and/or genetic rescue	References
*Pi3k iSH2-p110*α (GOF)	E15.5	E18.5: M	–	–	–	–	Konno et al., 2005
*Pi3k WT*	E14.5	E18.5: M	–	–	–	–	Baek et al., 2015
*Pi3k E545K (GOF)	E14.5	E18.5: M, C	–	–	–	–	Baek et al., 2015
*Pi3k E545K (GOF)	E14–E15, SSC	–	P15–70 L2/3 PN: No change-RMP No change-R_input_ No change-I/O (rheobase) No change-AP voltage threshold	–	–	–	Goz et al., 2020
*Pi3k E545K (GOF)	E14.5, SSC, CC	P24–28: C, D P60: M, C	P24–28, L2/3 PN: ↓ mIPSC frequency ↓ mIPSC amplitude	Spontaneous seizures	P60: ↓ GABAergic interneuron density	**Rapamycin**, 2 mg/kg every 48 h, P10–P30: rescued C, D, mIPSC frequency; partially rescued GABAergic interneuron density; no rescue of M, mIPSC amplitude	Zhong et al., 2021
*Pten* (CRISPR/Cas9 KO)	E14–E15 (rat)	P19: M, C, D	P21–P30, L2/3 PN: ↑ mEPSC frequency ↑ sEPSC frequency No change-RMP ↓ R_input_	–	–	–	Chen et al., 2015
*Akt1 WT*	E14.5	E16.5, E17.5, E18.5: enhanced migration	–	–	–	–	Itoh et al., 2016
*Akt1 m*Δ*PH* (GOF)	E14.5	E16.5, E17.5: M	–	–	–	–	Itoh et al., 2016
*Akt3 WT*	E14.5	E18.5: M, C P20: M, C	–	∼P28: Spontaneous bursts		**Rapamycin**, 3 mg/kg daily, E15.5–E18.5: rescued M, C	Baek et al., 2015
*Akt3 E17K (GOF)	E14.5	E18.5: M, C P20: M, C	–	∼P28: Spontaneous seizures	No microglia reactivity	**Rapamycin**, 3 mg/kg daily, E15.5–E18.5: rescued M, C **Rapamycin**, 3 mg/kg daily, P1–P3: no rescue of M ***Reelin*** **siRNA** or ***Foxg1 T271A*** **(LOF) expression**: partially rescued M	Baek et al., 2015
*Akt3 S472E* (GOF)	E14.5	E18.5: M	–	–	–	–	Baek et al., 2015
*Tsc1*^floxed/mutant^, Cre IUE, 2-hit model	E15–16, SSC	P15: C P28: M, C	–	P15: ↓ Seizure threshold	P15, P28: No astroglial reactivity	–	Feliciano et al., 2011
*Tsc1* (CRISPR/Cas9 KO)	E14	>P21: M, C	–	>P21: Spontaneous seizures	–	–	Lim et al., 2017
*Tsc1* (CRISPR/Cas9 KO)	E14–E15, SSC	–	P15–70 L2/3 PN: ↓ RMP (hyperpolarized) ↓ R_input_ ↓ I/O (=↑ rheobase) ↓ AP voltage threshold	–	–	–	Goz et al., 2020
*Tsc2* (shRNA KD)	E14	E19: M, C	–	–	–	**Rapamycin**, 5 mg/kg daily, E15–E18: rescued M, C	Tsai et al., 2014
*Tsc2* (shRNA KD)	E13.5, E16.5	E18.5, P2: M	–	–	–	***Cul5*** **shRNA KD**: rescued M	Moon et al., 2015
*Tsc2* (CRISPR/Cas9 KO)	E14	E18: M >P21: M, C	–	>P21: Spontaneous seizures	–	**Rapamycin**, 10 mg/kg daily, starting after seizure onset: rescued C; ↓ seizures	Lim et al., 2017
*Rheb WT*	E13.5, E14.5, E16.5	E18.5, P0, P2: M	–	–	–	***Cul5* shRNA KD**: rescued M	Moon et al., 2015
*Rheb WT*	E14.5, SSC	P0, P7: M	–	>P20: Spontaneous seizures	–	–	Reijnders et al., 2017
*Rheb WT*	E14.5–E16, mPFC, SSC	P14: M, C, D	–	–	–	–	Sokolov et al., 2018
*Rheb WT*	E14.5	–	–	No seizures	–	–	Zhao et al., 2019
*Rheb S16H* (GOF)	E15, mPFC	P8: M	–	–	P0: ↑ Axon growth	***4EBP1 F113A* (GOF) expression, *S6K1/2* shRNA KD**, or **lithium chloride (GSK3 blocker)**, 10 mg/kg daily, E15–E19: rescued axon overgrowth **GSK3β^DN^ expression**: rescued axon overgrowth; no rescue of M	Gong et al., 2015
*Rheb S16H* (GOF)	E15.5, mPFC	P0, P7, P28: M P24: C P28–P42: D	P28–P42 L2/3 PN: ↓ Spine density ↓ sEPSC frequency ↑ RMP (depolarized)	–	P7, P21–28: ↓ Autophagy	***4EBP1 F113A* (GOF) expression**: rescued M, C; partially rescued D; restored RMP, sEPSC frequency; no rescue of spine density ***S6K1/2* shRNA KD**: no rescue of M	Lin et al., 2016
*Rheb S16H*, conditional (+DCX-Cre; expression in migrating neurons)	E15.5, mPFC	P7: M	–	–	–	^–^	Lin et al., 2016
*Rheb S16H* (GOF)	E15.5, mPFC	P28: C, D >P56: M	–	>P56: Spontaneous seizures	>P56: ↑ Astroglial reactivity No change in GABAergic interneuron density	**Rapamycin**, 1 mg/kg every 48 h, P1–P56: rescued M, C; ↓ seizures	Hsieh et al., 2016
*Rheb S16H*, conditional (+tamoxifen-inducible Cre; postnatal expression)	E15.5, mPFC *P7 tamoxifen treatment	>P56: C, no M	–	>P56: Spontaneous seizures	–	–	Hsieh et al., 2016
*Rheb S16H* (GOF)	E15, mPFC	>P56: M, C	–	>P56: Spontaneous seizures	>P56: ↑ Microglial reactivity	–	Nguyen et al., 2019
*Rheb S16H* (GOF)	E15, SSC	P14: M, C, D	–	–	P14: ↑ Vascular density	**Rapamycin**, 0.5 mg/kg every 48 h, P1–P14: rescued D; partially rescued M, C; ↓ vascular density	Zhang et al., 2019
*Rheb S16H* (GOF)	E15, mPFC	>P28: M, C, D	–	>P42 Spontaneous seizures	–	***Flna* shRNA KD**: partially rescued M, C, D; ↓ seizures **PTI-125 (Flna modulator)**, 6 or 12 mg/kg 2× daily, P8–28: partially rescued C, D **PTI-125 (Flna modulator)**, 12 mg/kg 2× daily, P8–65: ↓ seizures; no rescue of M **PTI-125**, 12 mg/kg 2× daily, P29–54: partially rescued C, D; ↓ seizures	Zhang et al., 2020
*Rheb S16H* (GOF)	E15.5, mPFC	>P84: M, C	P8–12, L2/3 PN: ↑ h current P26–42, L2/3 PN: ↑ RMP (depolarized) ↓ R_input_ ↓ I/O ( = ↑ rheobase) ↑ h current (and ectopic HCN4 expression) ↑ Sag response	>P84: Spontaneous seizures	–	**Rapamycin**, 1 mg/kg every 48h, P1–P56: ↓ HCN4 expression ***Kir2.1* expression**: ↓ RMP, ↓ I/O, ↓ seizures; no rescue of C, capacitance ***HCN4***^*NF*^ **expression**: ↓ RMP, ↓ h current, ↓ sag response, ↓ seizures; no rescue of C, I/O	Hsieh et al., 2020
**Rheb P37L (GOF)	E14.5, SSC	P0, P7: M	–	>P20: Spontaneous seizures	–	–	Reijnders et al., 2017
**Rheb P37L (GOF)	E14.5, SSC	P30: M, C P25: D	P21–25, L2/3 PN: No change-RMP ↑ Capacitance ↓ R_membrane_ ( = ↓ R_input_) ↓ I/O ( = ↑ rheobase) No change-AP voltage thresholdP21–25, contralateral, non-transfected L2/3 PN targeted by mutant axons: ↑ I/O, ↑ eEPSC amplitude in response to depolarizing mutant axons	>P21: Spontaneous seizures	P45: ↑ Axon growth	**Rapamycin**, 1 mg/kg daily, E15.5-E16.5: partially rescued M; no rescue of seizures**Rapamycin**, 10 mg/kg daily, starting after seizure onset for 7 days: ↓ seizures ***Rheb P37L* deletion** (before seizure onset at P14 or after seizure onset): ↓ seizures; no rescue of M ***Tetanus toxin light chain*** **expression** (blocks axonal projection): prevented seizures ***Tetanus toxin light chain*** **expression** (before seizure onset at P14; blocks vesicular release): prevented seizures, I/O changes in contralateral neurons targeted by Rheb ***Tetanus toxin light chain*** **expression** (after seizure onset at P35; blocks vesicular release): ↓ seizures	Onori et al., 2020
*Rheb P37L*, conditional (+ tamoxifen-inducible Cre; postnatal expression)	E14.5, SSC *P7 or P21 tamoxifen treatment	No M	–	>P35: Spontaneous seizures	–	–	Onori et al., 2020
**Rheb S68P (GOF)	E14.5, SSC	P0, P7: M	–	>P20: Spontaneous seizures	–	–	Reijnders et al., 2017
*Rheb Y35L (GOF)	E14.5	E18.5: M, C P30: C	–	>P30: Spontaneous seizures	–	**Rapamycin**, 10 mg/kg daily, starting at P45 for 11 days: ↓ seizures	Zhao et al., 2019
*mTOR WT*	E14.5	P0: No M, C	–	–	–	*–*	Kassai et al., 2014
*mTOR WT*	E14	–	–	No seizures	–	–	Lim et al., 2015; Park et al., 2018; Kim et al., 2019
*mTOR WT*	E15 (rat)	E20: no M	–	–	–	*–*	Pelorosso et al., 2019
*mTOR SL1* + *IT* (GOF)	E14.5	P0: M, C	–	–	–	**Rapamycin**, 5 mg/kg, embryonic, or **Raptor shRNA KD**: rescued M, C **S6K1/2 shRNA KD**: rescued C	Kassai et al., 2014
*mTOR SL1* + *IT* (GOF)	E13.5, SSC	E17.5: M	–	–	–	–	Tarkowski et al., 2019
*mTOR L2427P (GOF)	E14	E18: M >P21: C	–	>P21: Spontaneous seizures	–	**Rapamycin**, 10 mg/kg daily, starting after seizure onset for 2 weeks: rescued C; ↓ seizures	Lim et al., 2015
*mTOR L2427P (GOF)	E14	–	–	–	>P56: Defective ciliogenesis ↓ Autophagy	**Rapamycin**, 10 mg/kg daily, starting after seizure onset for 2 weeks: rescued ciliogenesis	Park et al., 2018
*mTOR L2427P (GOF)	E14	E18, P7: M P21: C	P21, L2/3 PN: ↓ Spine density	>P21: Spontaneous seizures	E18: Translational dysregulation	**Rapamycin**, 5 mg/kg daily, E14–E17: rescued M **eIF4E shRNA KD**: rescued M, C, spine density; ↓ seizures **Metformin (eIF4E inhibitor)**, 200 mg/kg daily, P14–56: rescued C; ↓ seizures **Metformin**, 200 mg/kg daily, P84–114: ↓ seizures **ADK shRNA KD** or **5-ITU (ADK inhibitor)**, 1 or 2.6 mg/kg, 2×xdaily for 10 days: ↓ seizures	Kim et al., 2019
*mTOR A1459D (GOF)	E14.5	E18.5: M, C	–	–	–	–	Hanai et al., 2017
*mTOR C1483Y (GOF)	E14	E16, E18: M >P56: C	–	>P21: Spontaneous seizures	>P56: Defective ciliogenesis ↓ Autophagy	**Rapamycin**, 10 mg/kg daily, after seizure onset for 2 weeks: rescued C, ciliogenesis defects **Ofd1 shRNA KD**: rescued M, ciliogenesis defects; no rescue of C, seizures **Wnt5a expression**: rescued M	Park et al., 2018
*mTOR C1483Y (GOF)	E14	E18, P7: M P21: C	P21, L2/3 PN: ↓ Spine density	>P21: Spontaneous seizures	E18: Translational dysregulation	**Rapamycin**, 5 mg/kg daily, E14–E17: rescued M **eIF4E shRNA KD**: rescued M, C, spine density; ↓ seizures **Metformin (eIF4E inhibitor)**, 200 mg/kg daily, P14–56: rescued C; ↓ seizures **Metformin**, 200 mg/kg daily, P84–114: ↓ seizures **ADK shRNA KD** or **5-ITU (ADK inhibitor)**, 1 or 2.6 mg/kg, 2× daily for 10 days: ↓ seizures	Kim et al., 2019
*mTOR C1483Y (GOF)	E13.5, SSC	E17.5: M, C	–	–	–	–	Tarkowski et al., 2019
*mTOR L1460P (GOF)	E13.5, SSC	E17.5: M, C	–	–	–	–	Tarkowski et al., 2019
*mTOR S2215Y (GOF)	E13.5, SSC	E17.5: M, C	–	–	–	–	Tarkowski et al., 2019
*mTOR R2505P* (GOF)	E13.5, SSC	E17.5: M, C	–	–	–	–	Tarkowski et al., 2019
*mTOR L2427T* (GOF)	E13.5, SSC	E17.5: M	–	–	–	–	Tarkowski et al., 2019
*mTOR S2215F (GOF)	E15 (rat)	E20: M P28: C	–	–	–	–	Pelorosso et al., 2019
*Strad*α (shRNA KD)	E14	E17, E19: M	–	–	–	–	Orlova et al., 2010
*Strad*α (shRNA KD)	E14	E19: M	–	–	–	**Rapamycin**, 5 mg/kg daily, E15–E19: rescued M	Parker et al., 2013
*Depdc5* (CRISPR/Cas9 KO)	E14.5	E18.5: M P21–P63: M, C P20–P24: D	P20–P24, L2/3 PN: No change-Spine density ↑ Spine head width No change-sEPSC frequency ↑ sEPSC amplitude ↑ Capacitance ↓ R_input_ ↓ I/O (=↑ rheobase)	>P21: Spontaneous seizures	–	**Rapamycin**, 1 mg/kg single injection at E15: rescued M	Ribierre et al., 2018
*Depdc5* (CRISPR/Cas9 KO)	E13–E14 (rat)	P21–30: C	P21–28, L2/3 PN: No change-RMP ↓ R_input_ Doublet AP firing	>P60: Spontaneous seizures	–	**Everolimus**, P10–21: rescued C	Hu et al., 2018
*Depdc5*^floxed/mutant^, Cre IUE, 2-hit model	E14.5	P15: C P42: M, C, D	–	P42: ↓ Seizure threshold	–	***Depdc5 WT*** or ***Depdc5 Q542P*** **(GOF) expression**: rescued C ***Depdc5*** ***F164 del*** **(LOF)** **expression**: no rescue of C	Dawson et al., 2020
*Nprl3* (CRISPR/Cas9 KO)	E14	P3: M, C >P35: C	–	>P35: ↑ Cortical excitability ↓ Seizure threshold	–	**Rapamycin**, 1 mg/kg single injection at E15: rescued M, C	Iffland et al., 2020

*The table is organized by position of genes in the PI3K-mTOR pathway and GATOR1 complex, variant, and date of publication. All gene variants activate mTORC1 signaling. Stars in front of gene name denote human de novo somatic (^∗^) or germline (^∗∗^) mutations that have been identified in MCD (e.g., HME, FCDII) and epilepsy. All studies were done in mice unless noted otherwise. Targeted cortical areas are listed if they were specified in the original publication. Ages refers to when IUE was performed and the timepoints of pathological and/or behavioral evaluation. GOF, gain-of-function; LOF, loss-of-function; WT, wildtype; KO, knockout; KD, knockdown; E, embryonic day; P, postnatal day; SSC, somatosensory cortex; CC, cingulate cortex; mPFC, medial prefrontal cortex; L2/3 PN, layer 2/3 pyramidal neurons; RMP, resting membrane potential; R_input_, input resistance; R_membrane_, membrane resistance; I/O, input/output; mIPSC, miniature inhibitory postsynaptic current; mEPSC, miniature excitatory postsynaptic current; sEPSC, spontaneous excitatory postsynaptic current; eEPSC, evoked excitatory postsynaptic current; AP, action potential; DN, dominant negative; NF, non-functional.*

## Pi3K-mTor and Gator1 Gene Variants Resulting in Malformation of Cortical Development and Epilepsy

Early evidence for aberrant mTORC1 activation in TSC, FCDII, and HME, was identified by histological analysis of brain specimens from patients who underwent surgery for epilepsy (Baybis et al., 2004; Miyata et al., 2004; Ljungberg et al., 2006; Aronica et al., 2007). mTORC1 hyperactivation was also later demonstrated in patients with the rare neurodevelopmental disorder, PMSE (Puffenberger et al., 2007). These disorders collectively termed mTORopathies, share key pathological hallmarks, including MCD with cortical enlargement, mislamination, gliosis, and the presence of dysmorphic, cytomegalic neurons (Crino, 2015). TSC, FCDII, and HME are primarily distinguished based on the extent of the affected cortical regions, with HME having broader MCDs involving as much as whole cerebral hemispheres while TSC and FCDII are characterized by focal MCDs. These mTORopathies are associated with frequent seizures and are the most common causes for childhood intractable epilepsy (Harvey et al., 2008; Crino, 2015).

The mTOR pathway is a ubiquitously expressed signaling pathway that is involved in many important cellular processes, including cell growth, protein synthesis, autophagy, metabolism, and lysosome biogenesis (Saxton and Sabatini, 2017). In the brain, mTOR signaling controls cortical development and neural-specific functions, such as synaptic plasticity and learning and memory (Lipton and Sahin, 2014; Takei and Nawa, 2014). As a result, dysregulation of mTOR signaling has been implicated in several neurodevelopmental and neuropsychiatric disorders (Costa-Mattioli and Monteggia, 2013). The mTOR protein is an evolutionary conserved serine/threonine kinase. Association of mTOR with the Raptor adaptor protein characterizes mTORC1, a rapamycin-sensitive complex that regulates protein synthesis via direct phosphorylation of the translational regulators p70 S6 kinase 1 and 2 (S6K1/2) and eIF4E-binding protein 1 and 2 (4E-BP1/2) (Ma and Blenis, 2009; Saxton and Sabatini, 2017). Levels of phosphorylated S6K1/2 [or its downstream effector, ribosomal protein S6 (RPS6)] and 4E-BP1/2 are reliably used as readouts for mTORC1 activity. mTORC1 regulation of autophagy and metabolism occurs via additional downstream effectors (Saxton and Sabatini, 2017).

The best-characterized upstream regulation of mTORC1 activity is through the PI3K-AKT signaling pathway (Dibble and Cantley, 2015). PI3K is a lipid kinase that is activated by cell-surface receptors in response to extracellular cues such as growth factors. PI3K activates AKT, which directly phosphorylates and inactivates the TSC1/TSC2 complex. Inactivation of TSC1/2, which functions as GTPase-activating proteins (GAPs), results in activation of the GTP-binding protein RHEB that in turn activates mTORC1. The lipid phosphatase PTEN works as a negative regulator of mTORC1 signaling by counteracting PI3K function and thereby opposing downstream activation of mTORC1. In addition to PI3K-AKT signaling, recent studies have identified the GATOR1 complex, which consists of DEP domain containing 5 (DEPDC5) and the Nitrogen permease regulator 2-like (NRPL2) and 3-like (NPRL3) proteins, as a negative regulator of mTORC1 activity in response to cellular amino acid levels (Bar-Peled and Sabatini, 2014). GATOR1 functions as GAPs that inhibit mTORC1 activity by inactivating the Rag GTPases. When amino acid levels are low, GATOR1 signals to inhibit mTORC1. Conversely, when amino acid levels are adequate, GATOR1 releases mTORC1 inhibition to promote cell growth. Thus, there are two major molecular branches, PI3K/PTEN-AKT-TSC-RHEB and GATOR1, that control mTORC1 activity (Figure 1).

Genetic mutations leading to mTORC1 hyperactivity were first identified in the prototypical mTORopathy, TSC. Individuals with TSC have germline and somatic inactivating mutations in the *TSC1* or *TSC2* genes, leading to mTORC1 activation in multiple organs, including the brain (Kwiatkowski, 2003; Orlova and Crino, 2010). More recently, the emergence of advanced sequencing techniques led to the seminal discovery that FCDII and HME are caused by *de novo* brain somatic mutations in mTORC1 pathway genes that occur during neurodevelopment. Work by Lee et al. (2012) and Poduri et al. (2012) was first to demonstrate that brain somatic mutations in *PI3K*, *AKT3*, and *mTOR* are causal of HME. This was followed by crucial studies from Lim et al. (2015) and Nakashima et al. (2015)

showing that brain somatic mutations in *mTOR* cause FCDII. Brain somatic mutations in *TSC1* or *TSC2*, in the absence of germline mutations, also lead to FCDII (Lim et al., 2017). Numerous studies have been published since and have been discussed in several review articles (Marsan and Baulac, 2018; Muhlebner et al., 2019). PMSE is caused by a homozygous deletion in the *STE20-related kinase adaptor*α (*Strada*) gene, a modulator of mTORC1 signaling via the AMP-activated protein kinase (AMPK)-TSC1/2 pathway (Puffenberger et al., 2007; Orlova et al., 2010). To date, pathogenic variants in 14 distinct genes along the PI3K-mTOR pathway and GATOR1 complex, including *PI3K*, *PTEN*, *AKT3*, *TSC1*, *TSC2*, *RHEB*, *MTOR*, *STRADA*, *DEPDC5*, *NPRL2*, *NPRL3*, *KPTN*, *SZT2*, and TBC1D7, have been linked to MCD and epilepsy (Kim and Lee, 2019; Crino, 2020). Mutations in some genes (i.e., *MTOR*) occur much more frequently than in others (i.e., *KPTN*, *SZT2*, and *TBC1D7*) (D’Gama et al., 2017; Baldassari et al., 2019; Sim et al., 2019). Given that all of these mutations converge on mTORC1 leading to its hyperactivation, pharmacological intervention with mTOR inhibitors (e.g., rapamycin and its analog everolimus) has been proposed as a therapeutic option for epilepsy associated with these MCDs. In 2018, the rapamycin analog everolimus was US Food and Drug Administration (FDA)-approved to treat seizures in individuals with TSC (French et al., 2016). The efficacy of everolimus in controlling seizures in individuals with FCDII is currently assessed in a clinical trial (ClinicalTrials.gov identifier: NCT03198949).

## Designing Animal Models of mTORopathies

Hyperactive mTORC1 signaling leads to cellular and network alterations resulting in MCD and epilepsy (Lasarge and Danzer, 2014; Represa, 2019). However, the precise mechanisms remain elusive. Many animal models of mTORopathies have been established to aid in understanding the impact of mTORC1 hyperactivation on brain development and epilepsy. Brain-specific, conditional transgenic mice, such as *Pten*, *Tsc1*, *Tsc2*, and *Depdc5* knockout and *mTOR* gain-of-function mutation, exhibit key human mTORopathy phenotypes, including enlarged cortex, ectopic neuronal placement, neuronal hypertrophy, gliosis, and epilepsy (e.g., Kassai et al., 2014; Ostendorf and Wong, 2015; Yuskaitis et al., 2018). By contrast, global homozygous transgenic mice with constitutive mTOR activation are embryonic lethal. Newer rodent models using IUE to focally express or suppress mTORC1 pathway regulating genes in the cortex (discussed more below), as well as *Tsc2* and *Depdc5* zebrafish models (Kim S. H. et al., 2011; de Calbiac et al., 2018; Swaminathan et al., 2018), also recapitulate many of the mTORopathy phenotypes. Recent models also include patient-derived induced pluripotent stem cells (IPSCs) and cortical organoids, which provide the advantage of studying mTOR function in human cell populations (Blair et al., 2018; Andrews et al., 2020; Dang et al., 2020; Eichmüller et al., 2020). Both transgenic and IUE-based animal models as well as *in vitro* models have been vital for mechanistic and preclinical drug studies.

**FIGURE 2:**
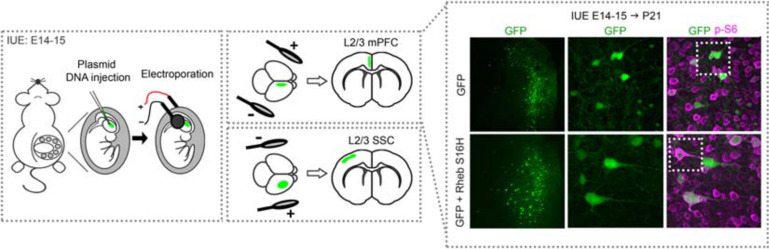
Schematic diagram of *in utero* electroporation and example of electroporated cortices. Rodents are electroporated at E14–15 (±0.5), which targets L2/3 cortical pyramidal neurons. Briefly, plasmid DNA is injected into the lateral ventricles and electrical pulses are passed through to electroporate the plasmid into neural progenitor cells (i.e., radial glia). The position of the electrodes that generate the pulses direct the plasmid into the specific brain region of interest, e.g., mPFC or SSC. An example of cortices from mice electroporated with GFP or GFP + *Rheb S16H* plasmids in the mPFC are shown. Note that GFP-expressing cells are positioned in L2/3 while GFP + *Rheb S16H*-expressing cells are misplaced across the cortical layers. GFP + *Rheb S16H*-expressing cells are also larger and exhibit increased phospho (p)-S6 levels, a marker of mTORC1 activity. *E, embryonic day; L, layer; mPFC, medial prefrontal cortex; SSC, somatosensory cortex.*

The MCD pathology in TSC, FCDII, and HME are thought to be caused by constitutive mTORC1 hyperactivation in early neuroglial progenitor cells during embryonic development, leading to a subset of affected neurons within a diffuse or localized area of the cortex (Poduri et al., 2013; Prabowo et al., 2013; Tsai et al., 2014; D’Gama et al., 2017). It is thought that the timing of when the somatic mutations occur determines the extent of the affected brain region, such that mutations leading to broader MCDs in HME occur earlier in development while those leading to focal MCDs in TSC and FCDII occur later in development (D’Gama et al., 2017). Additionally, it has been proposed that somatic mutations occurring early on, before hemispheric cleavage, can lead to bilateral asymmetric FCDII, where one hemisphere display apparent focal MCDs whereas the other side does not have visible malformations despite containing mutated cells, due to an uneven hemispheric distribution of mutation load (Guerrini et al., 2021). While transgenic mouse models have been important in studies of global MCDs, models that more precisely recapitulate focal lesions in TSC and FCDII provide additional advantages. One such TSC mouse model combined exogenous injection of a Cre-expressing viral vector in *Tsc1*^*floxed/floxed*^ pups. By controlling the dose of the viral vector, investigators were able to achieve mosaic *Tsc1* loss in a localized region similar to those observed in human TSC (Prabhakar et al., 2013). Another approach that has emerged within the last decade is based on IUE. This technique allows for *in vivo* manipulation of specific cell types in select brain regions (LoTurco et al., 2009). Using this technique, plasmid DNA targeting PI3K-mTOR or GATOR1 components are microinjected into the ventricles of embryonic rodent brains and transfected via electrical pulses into neural progenitor cells (i.e., radial glia) that line the ventricles. The position of the electrodes that generate the electrical pulses, which is manually determined by the investigator, directs plasmid expression into the brain region of interest (Figure 2). Current IUE-based models of mTORopathies include expression of *Pi3k*, *Akt*, *Rheb*, and *mTOR* gain-of-function variants (mTORC1 activators) or suppression of *Pten*, *Tsc1, Tsc2*, *Strad*α, *Depdc5*, and *Nprl3* (mTORC1 negative regulators) expression (Table 1 and Figure 1). Some of the gain-of-function variants are mutations that have been identified in patients with MCD and epilepsy while others are experimental mutations predicted to be pathogenic. Suppression of the negative regulators is achieved by CRISPR/Cas9 gene editing, which leads to gene knockout, or short hairpin RNA (shRNA)-mediated methods, which leads to gene knockdown. Additionally, two studies used a 2-hit model where a Cre plasmid was expressed by IUE in *Tsc1^*floxedmutant*^ or Depdc5^*floxed/mutant*^* mice to drive cell-type and -region specificity. The studies reviewed here all targeted cortical pyramidal neurons of layer (L) 2/3 of either the somatosensory cortex (SSC) or medial prefrontal cortex (mPFC), as these principal cells are proposed to be most severely affected in MCDs (Blumcke et al., 2009; Hadjivassiliou et al., 2010; D’Gama et al., 2017). Expression in L2/3 pyramidal neurons is achieved by performing IUE at embryonic day (E) 14–15 (±0.5), which targets radial glia generating upper layer cortical neurons. It is important to note that although IUE targets radial glia that give rise to both neurons and astrocytes, episomal plasmids that are used in standard IUE will only be expressed in neurons due to a plasmid dilution effect that occurs in dividing cells (Feliciano et al., 2011; Chen and LoTurco, 2012). In contrast, CRISPR/Cas9 plasmids integrate into the genome of the electroporated radial glia and are expected to label all cell types in the radial glial cell lineage (i.e., neurons and astrocytes). Overall, in addition to providing region- and cell-specificity, IUE offers the flexibility to vary the type of gene variants expressed, the timing of plasmid expression, and the affected region in a relatively simple manner.

## Conserved mTorc1-Dependent Phenotypes Across Pi3K-mTor and Gator1 Gene Variants

Ectopic neuronal placement and the presence of dysmorphic, cytomegalic neurons are major histopathological findings in TSC and FCDII (Wong, 2008; Blumcke et al., 2011). All gene variants reviewed here (see Table 1) consistently reproduce these phenotypes, supporting the notion that the underlying mechanisms for these alterations involve the mTORC1 pathway and its downstream processes. Additional histopathological phenotypes, such as the presence of balloon cells, have not been reported in these studies. Gliosis, including astrocyte and microglia reactivity, is regularly observed but seems to track with seizure activity (Feliciano et al., 2011; Nguyen et al., 2019), and is not discussed here.

Cortical pyramidal neurons destined to L2/3 are generated from radial glia in the ventricular zone at E14–15 in mice. Newborn neurons migrate from the ventricular zone shortly after and reach their final position in the cortex by postnatal day (P) 7 (Greig et al., 2013; Kast and Levitt, 2019). Disruption in this process leads to ectopic neuronal placement and cortical mislamination. In line with this timeframe, IUE at E13.5–15.5 resulted in neuronal migration defects and misplacement in the SSC and mPFC for all evaluated gene variants (Table 1). Despite being misplaced in other cortical layers, these neurons preserved the molecular identity of L2/3 pyramidal neurons, as evidenced by positive staining for the upper layer markers Cux1 or Satb2 and negative staining for the deeper layer marker Ctip2 (Tsai et al., 2014; Baek et al., 2015; Moon et al., 2015; Lin et al., 2016; Park et al., 2018; Tarkowski et al., 2019; Iffland et al., 2020; Onori et al., 2020; Zhong et al., 2021). Prenatal treatment with rapamycin to reduce mTORC1 activity prevented the migration defects and led to correct placement of the neurons in the cortex, supporting that mTORC1 hyperactivity contributes to these defects (Parker et al., 2013; Kassai et al., 2014; Tsai et al., 2014; Baek et al., 2015; Ribierre et al., 2018; Kim et al., 2019; Iffland et al., 2020; Onori et al., 2020). Postnatal rapamycin treatment after P7, when cortical neuronal migration is complete, is not expected to rescue neuronal misplacement. Indeed, this expectation was confirmed in the *Pi3k E545* variant (Zhong et al., 2021). Additionally, two studies showed that mTORC1 hyperactivation once migration is complete, by using a conditional *Rheb S16H* or *Rheb P37L* plasmid with a tamoxifen-inducible Cre plasmid, had no effect on neuronal placement (Hsieh et al., 2016; Onori et al., 2020). Thus, the impact of mTORC1 hyperactivation on neuronal placement occurs during a specific developmental timeframe, and cortical mislamination can be prevented in a limited effectiveness window.

Neuronal cytomegaly was also consistently reported for the evaluated gene variants. Increased neuron soma size was reported as early as E17.5 (Tarkowski et al., 2019) and persisted into adulthood (Lim et al., 2015, 2017; Park et al., 2018; Ribierre et al., 2018; Nguyen et al., 2019; Zhang et al., 2019; Dawson et al., 2020; Hsieh et al., 2020; Zhong et al., 2021). Furthermore, increasing mTORC1 activity levels by varying concentration of the *Rheb S16H* plasmid resulted in dose-dependent increases in neuron soma size, supporting a direct relationship between mTORC1 activity level and soma size (Nguyen et al., 2019). Unlike with the migration defects, mTORC1-induced neuronal dysmorphogenesis is not restricted to an early developmental window, as hyperactivating mTORC1 signaling via conditional *Rheb S16H* expression at P7 resulted in enlarged neurons (Hsieh et al., 2016). Neuron soma size was reduced with both pre- and postnatal rapamycin (or everolimus) treatment (Kassai et al., 2014; Tsai et al., 2014; Baek et al., 2015; Lim et al., 2015, 2017; Hsieh et al., 2016; Hu et al., 2018; Park et al., 2018; Zhang et al., 2019; Iffland et al., 2020; Zhong et al., 2021). Moreover, postnatal rapamycin treatment reduced soma size regardless of whether treatment began in neonatal or adult ages, suggesting mTORC1 hyperactivation impact neuron size via a dynamic, modifiable process. Dendrite morphology was assessed in the IUE studies targeting *Pi3k, Pten, Rheb*, and *Depdc5*, and all revealed dendritic overgrowth involving (for some) increased dendrite thickness and complexity (Chen et al., 2015; Hsieh et al., 2016; Lin et al., 2016; Ribierre et al., 2018; Sokolov et al., 2018; Zhang et al., 2019, 2020; Dawson et al., 2020; Onori et al., 2020; Zhong et al., 2021). Rescue of dendrite morphology by postnatal rapamycin treatment was reported in the *Pi3k E545* and *Rheb S16H* variants, and rapamycin likely has the same effect for the other variants (Zhang et al., 2019; Zhong et al., 2021). Data on axons in the IUE models are more limited. One study reported accelerated axon growth in mPFC L2/3 neurons at P0 following *Rheb S16H* expression (Gong et al., 2015). Axon overgrowth to the contralateral cortex in SSC L2/3 neurons at P45 has also more recently been reported in mice expressing the *Rheb P37L* variant (Onori et al., 2020).

The effectiveness of rapamycin in restoring neuronal migration and morphology defects supports that these phenotypes are mTORC1-dependent processes. mTORC1 regulates several downstream intracellular pathways, which could contribute to these cytoarchitectural defects. We will discuss the following mTORC1-dependent mechanisms here: altered translational control via 4E-BP1/2 and S6K1/2, and defective neuronal ciliogenesis in the context of autophagy. We also briefly discuss mTORC1-mediated regulation of the reelin-disabled 1 (DAB1) signaling.

The best characterized function of mTORC1 is regulation of mRNA translation via direct phosphorylation of two major translational regulators, S6K1/2 and 4E-BP1/2 (Ma and Blenis, 2009). S6K1/2 controls translation initiation as well as other functions via a negative feedback loop through AKT (Manning, 2004). The only known function for 4E-BP1/2 so far is regulation of cap-dependent translation, whereby ribosome recruitment to mRNA is initiated via binding of the eukaryotic translation initiation factor 4F (eIF4F) complex to the mRNA 5′ cap structure (Richter and Sonenberg, 2005). 4E-BP1/2 serves as a translational repressor that inhibits eIF4F function. mTORC1 promotes translation by inhibiting 4E-BP1/2 activity, which disinhibits eIF4F (Figure 1). Studies by Lin et al. (2016) demonstrated that targeting 4E-BP1/2 via expression of a constitutive active *4E-BP1* mutant (*4E-BP1 F113A*), which resists inactivation by mTORC1, prevented *Rheb S16H*-induced neuronal misplacement. Furthermore, knockdown of *4E-BP2* alone, which leads to increased cap-dependent translation, led to ectopic neuronal placement (Lin et al., 2016). These findings support that increased cap-dependent translation is necessary and sufficient to induce neuronal misplacement. By contrast, targeting S6K1/2 did not rescue neuronal misplacement (Lin et al., 2016). The relevant role of cap-dependent translation in neuronal migration is corroborated by Kim et al. (2019) who demonstrated that knockdown of *eIF4E*, a component of the eIF4F complex, rescued misplacement in both the *mTOR L2427P* and *C1483Y* variants. With regards to neuronal morphology, expression of constitutive active *4E-BP1* or knockdown of *eIF4E* reduced soma enlargement caused by *Rheb S16H* and *mTOR L2427P* and *C1483Y*, respectively. Metformin, an inhibitor of cap-dependent translation, also reduced cell size in the two *mTOR* variants (Kim et al., 2019). Additionally, knockdown of S6K1/2 reduced the increased soma size associated *mTOR SL1* + *IT*, an experimental *mTOR* variant that harbors four point mutations, *V2198A*, *L2216H*, *L2260P*, and *I2017T* (Kassai et al., 2014). However, one study showed that expressing a wildtype or a gain-of-function mutant of RPS6, the canonical S6K1/2 effector, did not lead to migration defects or altered neuron soma size in rats, suggesting that dysregulation in this axis alone is not sufficient to induce these phenotypes (Pelorosso et al., 2019). In terms of axons, studies by Gong et al. (2015) showed that enhancing 4E-BP1 or reducing S6K1/2 activity prevented *Rheb S16H*-induced axon overgrowth *in vivo*, supporting *in vitro* studies demonstrating both 4E-BP1/2 and S6K1/2 involvements in axon development (Choi et al., 2008; Li et al., 2008; Morita and Sobue, 2009).

Since newborn neurons migrate along radial glia scaffolds to their final position in the cortex and IUE targets radial glia, failure of neurons to migrate properly in these IUE models could be due to defects in the radial glia scaffolding rather than in the neurons. To address this question, Lin et al. (2016) co-electroporated a conditional *Rheb S16H* plasmid with a doublecortin (DCX)-Cre plasmid to selectively express *Rheb S16H* in DCX-expressing migrating neurons. They found that mTORC1 hyperactivation in migrating neurons alone was sufficient to cause neuronal misplacement, supporting that mTORC1-induced neuronal migration defects can occur independently of radial glia (Lin et al., 2016). Nevertheless, it is conceivable that radial glia defects can aggravate this phenotype. Neuronal-based mechanisms for cell misplacement is further supported by work from Park et al. (2018) who demonstrated that neuronal autophagy-mediated primary cilia defects cause mTORC1-induced neuronal misplacement. The authors found that impaired autophagy following *mTOR L2427P and C1483Y* expression led to an accumulation of the OFD1 protein, a regulator of neuronal ciliogenesis, and disrupted cilia formation. Neuron cilia are essential organelles that integrate many important developmental signaling pathways, such as Wnt and Sonic hedgehog, and defective neuron cilia-mediated signaling can lead to abnormal migration and ectopic neuronal placement (Guemez-Gamboa et al., 2014). The authors showed that knockdown of *Ofd1* or expression of *Wnt5a* restored neuronal placement in the *mTOR C1483Y* condition, supporting that disrupted ciliogenesis underlies these alterations. Importantly, defective ciliogenesis was sensitive to rapamycin treatment in both the *mTOR C1483Y and mTOR C1483Y* conditions, verifying that this is an mTORC1-dependent mechanism that is potentially shared across the other gene variants. Another proposed mechanism for mTORC1-mediated migration defects is via the reelin-DAB1 signaling pathway. The reelin-DAB1 pathway controls cytoskeletal changes required for neuronal migration and is a well-known regulator of cortical lamination and cell positioning in the cortex (Forster et al., 2010; Frotscher, 2010). Reelin-DAB1 signaling is regulated by the ubiquitin E3 ligase, Cullin 5 (CUL5). Interestingly, studies by Moon et al. (2015) found that *Tsc2* knockdown or wildtype *Rheb* overexpression resulted in increased CUL5 expression, and reducing CUL5 abrogated the migration defects resulting from these variants. They further showed that rapamycin decreased CUL5 levels in a conditional *Tsc2*^–/–^ mouse model, suggesting that aberrant reelin- DAB1 signaling via CUL5 is an mTORC1-dependent mechanism for neuronal msiplacement observed in mTORopathies.

Overall, these IUE studies demonstrate that defects in neuronal migration and morphology are conserved phenotypes across distinct PI3K-mTOR and GATOR1 gene variants, and the mechanisms that underlie these alterations likely converge on mTORC1 and its downstream effectors. For example, cap-dependent translation was shown to be an important regulator of the migration and morphological phenotypes observed in the *Rheb* and *mTOR* variants, and it is likely that the other variants such as *Tsc1*, *Tsc2*, *Depdc5*, etc. also share these mechanisms. However, it is important to emphasize that while 4E-BP1/2 and eIF4E contribute to neuron size, other molecular players such as S6K1/2 may also be involved (Fingar et al., 2002). It is also important to recognize that distinct PI3K-mTOR and GATOR1 genes differentially activate other intracellular molecules, which can also contribute to the observed phenotypes independently of mTORC1. Some of these mTORC1-independent pathways are discussed more in the later sections. Lastly, while some targets are important for multiple phenotypes (e.g., 4E-BP1/2 on neuronal migration and morphology), other targets regulate specific pathological features. For example, targeting OFD1 rescued migration defects but not neuron size in the *mTOR* variants.

In the context of epilepsy, spontaneous seizures were reported in all studies that evaluated this phenotype. Postnatal rapamycin treatment, starting before or after seizure onset, sufficiently suppresses seizures in multiple models (Lim et al., 2015, 2017; Hsieh et al., 2016; Zhao et al., 2019; Onori et al., 2020). Interestingly, these studies consistently reported that mTORC1-induced seizures occur independently of neuronal placement, and preventing this alteration did not prevent seizures. Hsieh et al. (2016) and Onori et al. (2020) used a conditional *Rheb S16H* and *Rheb P37L* plasmid, respectively, to bypass the developmental window for neuronal migration and found that seizures still developed despite proper placement of the mutant neurons. Similarly, Park et al. (2018) showed that targeting the *Ofd1* gene responsible for disrupting neuronal ciliogenesis rescued cilia defects and neuronal misplacement but not seizures in the mTOR *C1483Y* condition. These findings support neuronal misplacement of mutant neurons is not necessary for seizure generation. It is nonetheless conceivable that neuronal misplacement renders seizures worse, but this has not been examined. In terms of neuronal morphology, increased neuron soma size, as well as altered dendritic complexity and axon length, are thought to impact neuronal integration into cortical circuits and thereby contribute to cortical hyperexcitability and network dysfunction that can lead to seizures and epilepsy (Lasarge and Danzer, 2014; Represa, 2019).

## Potential Shared and Divergent Sources of Cell-Autonomous Hyperexcitability

There is a large number of electrophysiological studies in *in vitro* (primarily in hippocampal neurons and some in cortical neurons) and transgenic mouse models of mTORopathies [for some examples, see: (Tavazoie et al., 2005; Wang et al., 2007; Bateup et al., 2011, 2013; Luikart et al., 2011; Weston et al., 2012; Xiong et al., 2012; De Fusco et al., 2020)]. These studies are not reviewed here, but they highlight gene-specific differences in neurons and tissue hyperexcitability. Findings of cell-autonomous changes in excitability came from original studies in human TSC and FCDII tissue samples. These studies reported that dysmorphic, cytomegalic neurons within the tissue samples exhibit altered intrinsic excitability that may contribute to the generation of epileptic activity (Cepeda et al., 2003, 2005, 2012; Wang et al., 2007). In addition, a recent IUE study in *Rheb S16H* mutant pyramidal neurons reported that decreasing neuronal excitability by expressing an inwardly-rectifying potassium channel (Kir2.1) significantly reduced seizures, supporting that mutant neurons are directly involved in seizure generation (Hsieh et al., 2020). In light of these findings supporting a cell autonomous mechanism of hyperexcitability, we review the electrophysiological data on neurons expressing the different PI3K-mTOR and GATOR1 gene variants. We note that non-cell autonomous mechanisms of hyperexcitability in the surrounding environment have been described in some of the reviewed studies (Zhang et al., 2019; Onori et al., 2020; Zhong et al., 2021), but there are not enough data to compare across gene variants and they will thus not be discussed.

Two main groups of functional parameters account for neuronal excitability: intrinsic biophysical properties [i.e., passive membrane properties and ion channel complements important for the generation of action potentials (AP)] and synaptic properties [i.e., dendritic spine properties and excitatory and inhibitory postsynaptic currents (EPSC and IPSC)]. These parameters have been examined by patch clamp electrophysiology recordings in acute brain slices in 9 IUE studies targeting *Pi3k, Pten, Tsc1, Rheb*, *mTOR*, and *Depdc5* (Table 1). While these variants display strikingly similar morphological phenotypes (as described above), the functional phenotypes are more variable. Most recordings were performed from L2/3 cortical pyramidal neurons in the SSC except for the *Rheb S16H* condition, which was performed in the mPFC. Of note, no single study examined all the above parameters at once and the age of recording was different for each study. The latter variable is particularly important for evaluating synaptic activity because under normal conditions, spine growth is complete by P21 and a period of autophagy-mediated spine elimination follows until P28 (White et al., 1997). A study in conditional *Tsc2*^±^ mice reported no changes in dendritic spine properties at P21 but found an increase in spine density at P28 (Tang et al., 2014). This was accounted for by the observation that *Tsc2*^±^ mice have decreased mTORC1-regulated autophagy, which reduced spine elimination but did not affect spine formation. Thus, age needs to be carefully considered when assessing synaptic activity.

Regardless of the differences in age of recording and cortical area (i.e., mPFC vs. SSC), a few biophysical changes were conserved across the gene variants. In particular, increased capacitance was found in mutant neurons expressing *Rheb P37L* (Onori et al., 2020) and *Depdc5* (Ribierre et al., 2018) variants, which is consistent with increased cell size. Additionally, *Pten*, *Tsc1*, *Rheb S16H*, *Rheb P37L*, and *Depdc5* mutant neurons all displayed decreased input resistance (R_in_) (Chen et al., 2015; Hu et al., 2018; Ribierre et al., 2018; Goz et al., 2020; Hsieh et al., 2020; Onori et al., 2020). As a result of decreased R_in_, mutant neurons required a larger current injection to reach AP threshold [i.e., decreased input/output (I/O) or increased rheobase]. These findings support a decreased firing responsiveness to excitatory inputs for these gene variants. Nevertheless, above the firing threshold, *Depdc5* mutant neurons were reported to fire an initial AP doublet (Hu et al., 2018) or displayed increased gain of firing frequency (i.e., generate more APs) (Ribierre et al., 2018), suggesting that once neurons reach their firing threshold, they are more excitable. These findings also suggest an alteration in the ion channel complement of the *Depdc5* mutant neurons. Interestingly, no changes in R_in_ or I/O curve were observed for neurons expressing the *Pi3k E545K* variant (Goz et al., 2020). Regarding the resting membrane potential (RMP), *Rheb S16H* mutant neurons in the mPFC displayed a depolarized RMP (Lin et al., 2016; Hsieh et al., 2020). In contrast, *Pi3k E545K, Pten, Rheb P37L, and Depdc5* mutant neurons in the SSC displayed no changes in RMP (Chen et al., 2015; Hu et al., 2018; Goz et al., 2020; Onori et al., 2020). *Tsc1* mutant neurons (by the CRISPR/Cas9 system) had hyperpolarized RMP when compared to control eGFP/mRFP-expressing neurons, although it is unknown whether this would also be the case when compared to control CRISPR/Cas9 neurons (Goz et al., 2020). It remains unclear why *Rheb S16H* and *Rheb P37L* variants have different effects on RMP. One possible reason is that *Rheb S16H* was expressed in the mPFC whereas *Rheb P37L* was expressed in the SSC. Since a neuron’s RMP is set by its ion channel complement, differentially expressed ion channels in mPFC vs. SSC *Rheb* mutant neurons may contribute to this difference, but this needs to be further explored. Although the complement of ion channels has not been thoroughly examined in these studies, one study has reported abnormal expression of an HCN channel isoform, HCN4 (discussed below) as a source of hyperexcitability (Hsieh et al., 2020).

With regards to synaptic function, the reported findings are highly variable and depend on the gene variant. *Pten* mutant neurons (at P21-P30) displayed increased miniature and spontaneous excitatory postsynaptic current (mEPSC and sEPSC, respectively) frequency (Chen et al., 2015), consistent with previous findings in conditional *Pten*^–/–^ mice (Williams et al., 2015). In contrast, *Rheb S16H* and *mTOR L2427P* and *C1483Y* mutant neurons (at P28–42 and P21, respectively) all displayed decreased spine density (Lin et al., 2016; Kim et al., 2019). *Rheb S16H* mutant neurons also had decreased sEPSC frequency. Similar decreases in cortical neuron spine density were observed in conditional *Tsc1*^–/+^ mice (Meikle et al., 2008). *Depdc5* mutant neurons (at P20–24) displayed no changes in spine density, but the size of the spine heads was increased, which was accompanied by an increase in sEPSC amplitude and no change in sEPSC frequency (Ribierre et al., 2018). This would likely result in an increased total excitatory charge. Only one study examined inhibitory postsynaptic currents (IPSCs), and it was reported that the frequency and amplitude of miniature IPSC (mIPSC) were decreased in *Pi3k E545K* mutant neurons (at P24–P28) (Zhong et al., 2021). These data suggest decreased inhibition onto the mutant neurons, assuming GABA_*A*_ receptor activation remains hyperpolarizing (Talos et al., 2012).

The diverging effects of *Pi3k, Pten, Tsc1, Rheb*, *mTOR*, and *Depdc5* variants on neuronal excitability, particularly synaptic activity, emphasize a critical need to systematically investigate the functional effects of these gene variants side-by-side in the same system (i.e., IUE), cell type, cortical region, and age to parse out the source of the differences (i.e., whether the observed effects are due to variant-specific functional differences giving rise to distinct effects or variable experimental conditions). Such side-by-side comparisons to investigate the effects of *Pten* and *Tsc1* knockout on hippocampal neurons have been performed *in vitro* by Weston et al. (2014). The authors found that *Pten* deletion led to an increase in both excitatory and inhibitory synaptic transmission, while *Tsc1* deletion reduced inhibitory but did not affect excitatory synaptic transmission within the same culture system. These findings support diverging effects of *Pten* and *Tsc1* on synaptic transmission, which may be accounted for by the differential impact of *Pten* and *Tsc1* deletion on AKT activation (discussed more in the next section). One consideration for the apparent differences in the functional phenotypes in the IUE studies is that different PI3K-mTOR and GATOR1 gene variants can lead to different levels of mTORC1 activation, which could contribute to variable phenotypes. Studies by Nguyen et al. (2019) showed that increasing concentration of the *Rheb S16H* plasmid during IUE led to dose-dependent increases in mTORC1 activation. Importantly, low concentrations of *Rheb S16H* did not result in seizures whereas higher concentrations of *Rheb S16H* resulted in robust seizure activity, emphasizing an mTORC1 activity level-dependent effect on these phenotypes. Thus, it will be important to carefully assess and compare the degree of mTORC1 activation with these variants. Finally, only a few studies have directly examined the dependence of mTORC1 activity on the above electrophysiological findings so far. In the *Pi3k E545K* mutant neurons, reducing mTORC1 activity with rapamycin treatment restored mIPSC frequency but not amplitude (Zhong et al., 2021). In *Rheb S16H* mutant neurons, neonatal rapamycin treatment eliminated aberrant HCN4 channel expression (Hsieh et al., 2020). Additionally, targeting mTORC1-regulated cap-dependent translation via expression of constitutive active 4E-BP1 restored RMP and sEPSC frequency, but it not did rescue spine density in these neurons (Lin et al., 2016). The reason for the discrepancy between sEPSC and spines data in *Rheb S16H* mutant neurons remains unclear. In contrast, knockdown of *eIF4E* to reduce cap-dependent translation rescued spine density in both *mTOR L2427P and C1483Y* mutant neurons. Given these few studies, the extent to which the observed functional phenotypes in the PI3K-mTOR and GATOR1 gene variants converge on mTORC1 and its downstream effectors remains unclear.

With regards to the source of epileptogenicity, all evaluated mutant neurons required higher depolarizations to generate APs as discussed above. How intrinsically more hypoexcitable neurons lead to cortical hyperexcitability and epilepsy remains unclear and is an active area of research. In *Pten* mutant neurons, increased excitatory neurotransmission might induce greater depolarization locally compared to control neurons, but it is unclear whether this is sufficient to counterbalance the low R_in_ to trigger firing (Chen et al., 2015). In *Depdc5* mutant neurons, once the AP threshold was reached, neurons displayed a higher firing gain (i.e., fire more APs) (Ribierre et al., 2018). Whether the combined increase in total excitatory charge and increased firing grain is sufficient to trigger the seizures observed in this model remains unknown. In *Rheb S16H* mutant neurons, abnormal expression of an HCN4 channel was found to be responsible for depolarized RMPs and repetitive firing in these neurons (Hsieh et al., 2020). Silencing HCN4 channel activity in *Rheb S16H* mutant neurons decreased RMPs and reduced the seizure frequency, thus providing a novel mechanism of mTORC1-induced hyperexcitability. HCN4 channels are highly sensitive to intracellular cAMP levels and do not depend on membrane depolarization for gating (Robinson and Siegelbaum, 2003; Brennan et al., 2016). Indeed, increasing intracellular cAMP levels by forskolin application depolarized *Rheb S16H* mutant neurons and led to repetitive AP firing (Hsieh et al., 2020), but how HCN4 channels are activated *in vivo* leading to seizures in *Rheb S16H* mice is unknown. Cortical neurons, including mPFC pyramidal neurons, receive dense noradrenergic projections from the locus coeruleus and dopaminergic projections from the ventral tegmental area (Chandler et al., 2014; Radnikow and Feldmeyer, 2018). Noradrenaline and dopamine released from these projections activate several receptors, including Gs-coupled β-adrenergic and D1/D5 dopaminergic receptors, which could contribute to cAMP increase and HCN4 channel activation in *Rheb S16H* mutant neurons (Grzelka et al., 2017; Radnikow and Feldmeyer, 2018). Nevertheless, this needs to be further investigated. Given that the abnormal expression of HCN4 in *Rheb S16H* mutant neurons is an mTORC1-dependent process (rapamycin treatment eliminated aberrant HCN4 expression) that can modulate epilepsy, it would be important to examine whether this phenotype is conserved across the different gene variants.

## Differential Activation of Intracellular Pathways in mTORopathies

While pathological variants in the PI3K-mTOR pathway and GATOR1 complex all lead to mTORC1 hyperactivity, they can also differentially modulate other intracellular signaling pathways independently of mTORC1. Here, we discuss several findings from the IUE studies reporting activation of parallel pathways by specific proteins in the PI3K-mTOR pathway: AKT-GSK3β, AKT- FOXG1-reelin, and TSC/RHEB-MAPK/ERK-FLNA (Figure 1). Other examples, such as the non-canonical function of TSC/RHEB on the notch signaling pathway, have been reviewed elsewhere (Neuman and Henske, 2011), although the function of mTORC1-independent pathways in mTORopathies overall remains understudied.

One of the best-established function of AKT, aside from regulation of cell growth via TSC1/2-RHEB-mTORC1 signaling, is phosphorylation and inhibition of the glycogen synthase GSK3β (Bellacosa et al., 2004). GSK3β regulates many neuronal processes, including neuronal polarization, axon growth, and axon branching (Kim Y. T. et al., 2011). Studies in the cancer field have shown that gain-of-function mutations in *Pi3k* and *Akt*, as well as *Pten* loss-of-function, upregulate AKT and lead to decreased GSK3β activity (Duda et al., 2020). By contrast, *Tsc1* loss-of-function or *Rheb* gain-of-function (genes downstream of AKT) indirectly downregulates AKT via a negative homeostatic feedback loop through mTORC1-S6K1/2, thereby increasing GSK3β activity (Shah et al., 2004; Meikle et al., 2008; Gong et al., 2015). It is expected that variants of the GATOR1 complex would lead to decreased AKT activity through the same feedback mechanism, but this needs to be examined. IUE-based studies by Gong et al. (2015) confirmed increased S6K1/2 activity and decreased GSK3β activity in mice expressing the *Rheb S16H* variant. Moreover, they found that decreasing GSK3β activity by expression of a dominant-negative GSK3β mutant prevented *Rheb S16H*-induced axon overgrowth *in vivo*. This effect was also observed when using the pharmacological GSK3β inhibitor, lithium chloride. Thus, one mechanism by which RHEB-induced mTORC1 hyperactivation causes axon dysmorphogenesis is via S6K1/2 feedback inhibition on AKT-GSK3β signaling. It remains unknown whether inhibiting GSK3β activity would also reduce axon overgrowth in neurons expressing other discussed variants downstream of AKT (e.g., *Tsc1, Tsc2*, and *mTOR*). Interestingly, mutant hippocampal neurons in conditional *Pten*^–/–^ mice with reduced GSK3β activity also displayed increased axon growth (Kwon et al., 2006), suggesting that too little or too much GSK3β activity leads to a similar axonal phenotype in hyperactive mTORC1 conditions.

Another described function of AKT is the regulation of the FOXG1 transcriptional repressor (Datta et al., 1999; Regad et al., 2007; Dastidar et al., 2011). FOXG1 plays crucial roles in early brain development, and loss of FOXG1 leads to microcephaly, developmental delay, and cerebral atrophy (Hettige and Ernst, 2019). Studies by Baek et al. (2015) showed that expression of the *Akt E17K* variant by IUE altered AKT-FOXG1 signaling in mice, resulting in increased reelin expression during development. They further showed that expressing a *Foxg1 T271A* mutant that resists modulation by AKT or reducing reelin via siRNA partially restored the migration deficits caused by *Akt3 E17K* expression. These findings suggest a role for AKT-FOXG1-reelin signaling in AKT-induced cortical mislamination, but it is unclear whether this is conserved across the other PI3K-mTOR or GATOR1 gene variants.

More recently, studies by Zhang et al. (2020) demonstrated increased levels of an actin cross-linking molecule, Filamin A (FLNA) following expression of *Rheb S16H* by IUE in mice. Histological analysis in resected human cortical tissue from individuals with FCDII confirmed increased FLNA expression in dysmorphic pyramidal neurons and balloon cells (Zhang et al., 2020). FLNA regulates cytoskeletal dynamics through interaction with multiple membrane proteins and plays important functions in neuronal migration (Feng and Walsh, 2004). Mutations in FLNA cause a neurodevelopmental disorder known as X-linked periventricular nodular heterotopia, which is associated with cortical malformation and seizures (Parrini et al., 2006). The authors showed that knockdown of FLNA prevented neuronal misplacement and reduced soma and dendrite hypertrophy in *Rheb S16H*-expressing mice, supporting a role for FLNA in cortical mislamination and neuronal dysmorphogenesis in mTORopathies. Aberrant neuronal morphology was also rescued using a pharmacological modulator of FLNA, PTI-125. More importantly, both FLNA knockdown and PTI-125 treatment reduced seizure activity in *Rheb S16H* mice, demonstrating a direct contribution of FLNA to epilepsy (Zhang et al., 2020). Interestingly, Zhang et al. (2020) showed that the *Rheb S16H*-induced FLNA increase in mice was insensitive to rapamycin treatment. Instead, inhibiting MAPK/ERK pathway, a parallel regulator of cell growth and development that is dysregulated in TSC (Maldonado et al., 2003; Magri et al., 2011; Chevere-Torres et al., 2012; Zhang et al., 2014), with PD0325901 treatment successfully reduced FLNA levels (Zhang et al., 2020). These findings revealed an mTORC1-independent process for *Rheb*-induced FLNA changes. Indeed, expressing the gain-of-function *mTOR* variants, *mTOR L1460P* and *S2215Y*, in cortical neurons *in vitro* did not lead to an increase in FLNA level (Zhang et al., 2020). Dysregulation of MAPK/ERK signaling and increased FLNA levels have also been reported in conditional *Tsc1*^–/–^ and *Tsc1*^±^ mice (Zhang et al., 2014, 2016). Thus, altered MAPK/ERK-FLNA function represents an mTORC1-independent mechanism that contributes to cortical malformations and epilepsy in TSC- and RHEB-related mTORopathies. It remains to be examined whether FLNA levels are increased in the other variants.

## Treatment for Human mTORopathies Beyond mTorc1 Inhibition

The crucial role of aberrant mTORC1 signaling in epilepsy is undebatable. The efficacy of rapamycin treatment in suppressing seizures has been demonstrated in numerous animal models of mTORopathies (Ostendorf and Wong, 2015), and the rapamycin analog, everolimus, has been approved for treating seizures in human TSC (French et al., 2016). The breakthrough usage of everolimus in clinics provides a new treatment option for intractable seizures in patients with TSC, however, everolimus is and remains the only mTORC1-targeting drug approved for epilepsy treatment. Given the growing body of genetic and molecular evidence for mTORC1 dysregulation in TSC, FCDII, and HME, there is a crucial need for novel treatment options that directly target the underlying molecular mechanisms.

Studies in the IUE-based models of mTORopathies that we review here provide some promising, potential molecular candidates for therapeutic intervention. Specifically, effective reduction of seizures was observed by modulating the activity of FLNA, HCN4, eIF4E, and adenosine kinase (ADK) in mice with mTORC1 hyperactivation. Work by Zhang et al. (2020) demonstrated that FLNA levels were aberrantly upregulated in dysmorphic neurons in patients with FCDII and mice expressing *Rheb S16H.* They further showed that knockdown of FLNA or treatment with an FLNA modulator, PTI-125, significantly reduced seizure activity by >60% in these mice (Zhang et al., 2020). Work by Hsieh et al. (2020) demonstrated that HCN4 was ectopically expressed in dysmorphicr neurons in patients in FCDII and TSC as well as mice expressing *Rheb S16H.* Silencing HCN4 activity by expression of a non-functional mutant form of HCN4 prevented epilepsy in these mice (Hsieh et al., 2020). Increased FLNA and HCN4 were found in the same *Rheb S16H* model and are mTORC1-independent and -dependent, respectively. Normalizing FLNA levels or blocking HCN4 activity reduced seizures, suggesting that both alterations contribute to seizures through different mechanisms and targeting one of these mechanisms is sufficient to reduce seizures. Work by Kim et al. (2019) provided evidence for aberrant translational regulation in patients with FCDII and mice expressing the patient variants *mTOR L2427P* and *C1483Y*. They showed that knockdown of the mTORC1-mediated translational regulator eIF4E or treatment with the eIF4E inhibitor, metformin, significantly decreased seizures in these mice. Kim et al. (2019) also showed that genetic or pharmacological inhibition of ADK, a novel downstream target of the mTORC1/eIF4F axis that was shown to be upregulated in patients with TSC, FCDII, and HME, alleviated seizures.

These studies are important because they show that while a combination of molecular and functional alterations is required for epileptogenesis, targeting a subset of these abnormalities is sufficient to reduce seizures. The strength of these studies is that they also reported abnormal expression of these potential targets in patients, establishing a clinical relevance for these molecules. FLNA and eIF4E can be pharmacologically targeted with available drugs, and therefore represent attractive targets. Both studies by Kim et al. (2019) and Zhang et al. (2020) showed that targeting FLNA or eIF4E, respectively, via drug treatment after seizure onset alleviated seizures, which is clinically important since seizures are often well established by the time patients are diagnosed with epilepsy and considered for treatment. Moreover, metformin is an FDA-approved drug to treat type 2 diabetes that could potentially be rapidly translated into clinical use for epilepsy. One limitation with targeting FLNA is that this is an mTORC1-independent mechanism (as discussed above), and thus may only apply for TSC and a subset of FCDII. As expected, PTI-125 treatment did not affect mTORC1 activity, suggesting that combination therapy with rapamycin may have added therapeutic benefits. Regarding HCN4, specific pharmacological inhibitors for these channels are not available and systematic inhibition of HCN4 function via a drug treatment would be unsafe given that HCN4 channels are responsible for the pacemaking activity of the heart (DiFrancesco, 2010). However, HCN4 could represent a potential molecular target for targeted gene therapies. Indeed, since focal MCDs, such as those observed in TSC and FCDII, are localized to specific areas in the brain, targeted gene delivery via direct brain injection could be an innovative treatment avenue for these mTORopathies.

## Conclusion

Studies using IUE-based models and others demonstrate that gene variants along the PI3K-mTOR signaling pathway and GATOR1 complex produce both shared and unshared phenotypes. These findings have significant clinical implications as they underscore a need to consider patient-specific variants when evaluating treatment strategies for intractable epilepsy. Functional (e.g., electrophysiology) and non-cell autonomous effects of these variants have been under-investigated and should be addressed in future studies as they will provide valuable insights into mechanisms of human pathology and disease etiology. As healthcare is moving toward precision medicine, we propose the idea of a targeted therapeutic strategy for epilepsy based on individual patient variants on a case-by-case basis. This would, however, require new diagnostic tools beyond neuroimaging and pathological examination of brain specimen to systematically detect the underlying gene variants and to visualize associated molecular changes.

## Author Contributions

Both authors listed have made a substantial, direct and intellectual contribution to the work, and approved it for publication.

## Conflict of Interest

LN and AB are co-inventors on one patent application, PCT/US2020/054007 entitled “Targeting Cap-Dependent Translation to Reduce Seizures in mTOR disorders.” AB is an inventor on two patent applications, PCT/US2020/020994 entitled “Methods of Treating and Diagnosing Epilepsy” and PCT/US2020/018136 entitled “Methods of Treating Epilepsy.”
