# Corrigendum: Convergent and Divergent Mechanisms of Epileptogenesis in mTORopathies

**DOI:** 10.3389/fnana.2021.715363

**Published:** 2021-07-06

**Authors:** Lena H. Nguyen, Angélique Bordey

**Affiliations:** ^1^Department of Neurosurgery, Yale School of Medicine, Yale University, New Haven, CT, United States; ^2^Department of Cellular & Molecular Physiology, Yale School of Medicine, Yale University, New Haven, CT, United States

**Keywords:** neuron migration, tuberous sclerosis complex, focal cortical dysplasia, GATOR1 complex, *in utero* electroporation, mTOR, cortical development, epilepsy

In the original article, there was a mistake in Table 1 as published.

Three rows of cited work (*Rheb S16H*, Hsieh et al., 2020; *Rheb P37L*, Reijnders et al., 2017, and *Rheb P37L*, Onori et al., 2020) were omitted.

Additionally, one of the cited studies (*Rheb S16H*) had the incorrect reference Zhang et al., 2019. Zhang, L., Huang, T., Teaw, S., and Bordey, A. (2019). Hypervascularization in mTOR-dependent focal and global cortical malformations displays differential rapamycin sensitivity. *Epilepsia* 60, 1255–1265. doi: 10.1111/epi.15969. Instead, it should be Zhang et al., 2020. Zhang, L., Huang, T., Teaw, S., Nguyen, L. H., Hsieh, L. S., Gong, X., et al. (2020). Filamin A inhibition reduces seizure activity in a mouse model of focal cortical malformations. *Sci. Transl. Med*. 12:eaay0289. doi: 10.1126/scitranslmed.aay0289.

The corrected Table 1 appears below.

**Table 1:** Summary of IUE-based rodent models of mTORopathies.

**Gene**	**IUE age, cortical area**	**Migration defect/misplacement (M), Cytomegaly (C), Dendrite overgrowth (D)**	**Synaptic function and electrophysiological properties**	**Seizure phenotype**	**Other phenotypes**	**Pharmacological and/or genetic rescue**	**References**
*Pi3k iSH2-p110α* (GOF)	E15.5	E18.5: M	–	–	–	–	Konno et al., 2005
*Pi3k WT*	E14.5	E18.5: M	–	–	–	–	Baek et al., 2015
*Pi3k E545K (GOF)	E14.5	E18.5: M, C	–	–	–	–	Baek et al., 2015
*Pi3k E545K (GOF)	E14-E15, SSC	–	P15-70 L2/3 PN: No change-RMP No change-R_input_No change-I/O (rheobase)No change-AP voltage threshold	–	–	–	Goz et al., 2020
*Pi3k E545K (GOF)	E14.5, SSC, CC	P24-28: C, DP60: M, C	P24-28, L2/3 PN: ↓ mIPSC frequency ↓ mIPSC amplitude	Spontaneous seizures	P60: ↓ GABAergic interneuron density	**Rapamycin**, 2 mg/kg every 48 h, P10-P30: rescued C, D, mIPSC frequency; partially rescued GABAergic interneuron density; no rescue of M, mIPSC amplitude	Zhong et al., 2021
*Pten* (CRISPR/Cas9 KO)	E14-E15 (rat)	P19: M, C, D	P21-P30, L2/3 PN: ↑ mEPSC frequency ↑ sEPSC frequencyNo change-RMP ↓ R_input_	–	–	–	Chen et al., 2015
*Akt1 WT*	E14.5	E16.5, E17.5, E18.5: enhanced migration	–	–	–	–	Itoh et al., 2016
*Akt1 mΔPH* (GOF)	E14.5	E16.5, E17.5: M	–	–	–	–	Itoh et al., 2016
*Akt3 WT*	E14.5	E18.5: M, CP20: M, C	–	~P28: Spontaneous bursts		**Rapamycin**, 3 mg/kg daily, E15.5-E18.5: rescued M, C	Baek et al., 2015
*Akt3 E17K (GOF)	E14.5	E18.5: M, CP20: M, C	–	~P28: Spontaneous seizures	No microglia reactivity	**Rapamycin**, 3 mg/kg daily, E15.5-E18.5: rescued M, C **Rapamycin**, 3 mg/kg daily, P1-P3: no rescue of M ***Reelin*** **siRNA** or ***Foxg1 T271A*** **(LOF) expression**: partially rescued M	Baek et al., 2015
*Akt3 S472E* (GOF)	E14.5	E18.5: M	–	–	–	–	Baek et al., 2015
*Tsc1*^floxed/mutant^,Cre IUE, 2-hit model	E15-16, SSC	P15: CP28: M, C	–	P15:↓ Seizure threshold	P15, P28: No astroglial reactivity	–	Feliciano et al., 2011
*Tsc1*(CRISPR/Cas9 KO)	E14	>P21: M, C	–	>P21: Spontaneous seizures	–	–	Lim et al., 2017
*Tsc1*(CRISPR/Cas9 KO)	E14-E15, SSC	–	P15-70 L2/3 PN: ↓ RMP (hyperpolarized) ↓ R_input_ ↓ I/O (= ↑ rheobase) ↓ AP voltage threshold	–	–	–	Goz et al., 2020
*Tsc2* (shRNA KD)	E14	E19: M, C	–	–	–	**Rapamycin**, 5 mg/kg daily, E15-E18: rescued M, C	Tsai et al., 2014
*Tsc2* (shRNA KD)	E13.5, E16.5	E18.5, P2: M	–	–	–	***Cul5*** **shRNA KD**: rescued M	Moon et al., 2015
*Tsc2*(CRISPR/Cas9 KO)	E14	E18: M>P21: M, C	–	>P21: Spontaneous seizures	–	**Rapamycin**, 10 mg/kg daily, starting after seizure onset: rescued C; ↓ seizures	Lim et al., 2017
*Rheb WT*	E13.5, E14.5, E16.5	E18.5, P0, P2: M	–	–	–	***Cul5*** **shRNA KD**: rescued M	Moon et al., 2015
*Rheb WT*	E14.5, SSC	P0, P7: M	–	>P20:Spontaneous seizures	–	–	Reijnders et al., 2017
*Rheb WT*	E14.5-E16, mPFC, SSC	P14: M, C, D	–	–	–	–	Sokolov et al., 2018
*Rheb WT*	E14.5	–	–	No seizures	–	–	Zhao et al., 2019
*Rheb S16H* (GOF)	E15, mPFC	P8: M	–	–	P0: ↑ Axon growth	***4EBP1 F113A*** **(GOF) expression**, ***S6K1/2*** **shRNA KD**, or **lithium chloride (GSK3 blocker)**, 10 mg/kg daily, E15-E19: rescued axon overgrowth **GSK3β^DN^** **expression**:rescued axon overgrowth; no rescue of M	Gong et al., 2015
*Rheb S16H* (GOF)	E15.5, mPFC	P0, P7, P28: MP24: CP28-P42: D	P28-P42 L2/3 PN: ↓ Spine density ↓ sEPSC frequency ↑ RMP (depolarized)	–	P7, P21-28: ↓ Autophagy	***4EBP1 F113A*** **(GOF) expression**: rescued M, C; partially rescued D; restored RMP, sEPSC frequency; no rescue of spine density ***S6K1/2*** **shRNA KD**:no rescue of M	Lin et al., 2016
*Rheb S16H*, conditional(+ DCX-Cre; expression in migrating neurons)	E15.5, mPFC	P7: M	–	–	–	–	Lin et al., 2016
*Rheb S16H* (GOF)	E15.5, mPFC	P28: C, D>P56: M	–	>P56: Spontaneous seizures	>P56: ↑ Astroglial reactivity No change in GABAergic interneuron density	**Rapamycin**, 1 mg/kg every 48h, P1-P56: rescued M, C; ↓ seizures	Hsieh et al., 2016
*Rheb S16H*, conditional(+ tamoxifen-inducible Cre; postnatal expression)	E15.5, mPFC *P7 tamoxifen treatment	>P56: C, no M	–	>P56: Spontaneous seizures	–	–	Hsieh et al., 2016
*Rheb S16H* (GOF)	E15, mPFC	>P56: M, C	–	>P56: Spontaneous seizures	>P56: ↑ Microglial reactivity	–	Nguyen et al., 2019
*Rheb S16H* (GOF)	E15, SSC	P14: M, C, D	–	–	P14: ↑ Vascular density	**Rapamycin**, 0.5 mg/kg every 48 h, P1-P14: rescued D; partially rescued M, C; ↓ vascular density	Zhang et al., 2019
*Rheb S16H* (GOF)	E15, mPFC	>P28: M, C, D	–	>P42Spontaneous seizures	–	***Flna*** **shRNA KD**: partially rescued M, C, D; ↓ seizures **PTI-125 (Flna modulator)**, 6 or 12 mg/kg 2x daily, P8-28: partially rescued C, D **PTI-125 (Flna modulator)**, 12 mg/kg 2x daily, P8-65: ↓ seizures; no rescue of M **PTI-125**, 12 mg/kg 2x daily, P29-54: partially rescued C, D; ↓ seizures	Zhang et al., 2020
*Rheb S16H* (GOF)	E15.5, mPFC	>P84: M, C	P8-12, L2/3 PN: ↑ h current P26-42, L2/3 PN: ↑ RMP (depolarized) ↓ R_input_ ↓ I/O (= ↑ rheobase) ↑ h current (and ectopic HCN4 expression) ↑ Sag response	>P84: Spontaneous seizures	–	**Rapamycin**, 1 mg/kg every 48h, P1-P56: ↓ HCN4 expression ***Kir2.1*** **expression:**↓ RMP, ↓ I/O, ↓ seizures; no rescue of C, capacitance ***HCN4**^***NF***^* **expression:**↓ RMP, ↓ h current, ↓ sag response, ↓ seizures; no rescue of C, I/O	Hsieh et al., 2020
**Rheb P37L (GOF)	E14.5, SSC	P0, P7: M	–	>P20:Spontaneous seizures	–	–	Reijnders et al., 2017
**Rheb P37L (GOF)	E14.5, SSC	P30: M, CP25: D	P21-25, L2/3 PN:No change-RMP ↑ Capacitance ↓ R_membrane_ (= ↓ R_input_) ↓ I/O (= ↑ rheobase) No change-AP voltage threshold P21-25, contralateral, non-transfected L2/3 PN targeted by mutant axons: ↑ I/O, ↑ eEPSC amplitude in response to depolarizing mutant axons	>P21:Spontaneous seizures	P45: ↑ Axon growth	**Rapamycin**, 1 mg/kg daily, E15.5-E16.5: partially rescued M; no rescue of seizures **Rapamycin**, 10 mg/kg daily, starting after seizure onset for 7 days: ↓ seizures ***Rheb P37L*** **deletion** (before seizure onset at P14 or after seizure onset): ↓ seizures; no rescue of M ***Tetanus toxin light chain*** **expression** (blocks axonal projection)**:** prevented seizures ***Tetanus toxin light chain*** **expression** (before seizure onset at P14; blocks vesicular release): prevented seizures, I/O changes in contralateral neurons targeted by Rheb ***Tetanus toxin light chain*** **expression** (after seizure onset at P35; blocks vesicular release): ↓ seizures	Onori et al., 2020
*Rheb P37L*, conditional(+ tamoxifen-inducible Cre; postnatal expression)	E14.5, SSC *P7 or P21 tamoxifen treatment	No M	–	>P35:Spontaneous seizures	–	–	Onori et al., 2020
**Rheb S68P (GOF)	E14.5, SSC	P0, P7: M	–	>P20:Spontaneous seizures	–	–	Reijnders et al., 2017
*Rheb Y35L (GOF)	E14.5	E18.5: M, CP30: C	–	>P30:Spontaneousseizures	–	**Rapamycin**, 10 mg/kg daily, starting at P45 for 11 days: ↓ seizures	Zhao et al., 2019
*mTOR WT*	E14.5	P0: No M, C	–	–	–	–	Kassai et al., 2014
*mTOR WT*	E14	–	–	No seizures	–	–	Lim et al., 2015; Park et al., 2018; Kim et al., 2019
*mTOR WT*	E15 (rat)	E20: no M	–	–	–	–	Pelorosso et al., 2019
*mTOR SL1+IT* (GOF*)*	E14.5	P0: M, C	–	–	–	**Rapamycin**, 5 mg/kg, embryonic, or **Raptor shRNA KD:** rescued M, C**S6K1/2 shRNA KD**: rescued C	Kassai et al., 2014
*mTOR SL1+IT* (GOF)	E13.5, SSC	E17.5: M	–	–	–	–	Tarkowski et al., 2019
*mTOR L2427P (GOF)	E14	E18: M>P21: C	–	>P21: Spontaneous seizures	–	**Rapamycin**, 10 mg/kg daily, starting after seizure onset for 2 weeks: rescued C; ↓ seizures	Lim et al., 2015
*mTOR L2427P (GOF)	E14	–	–	–	>P56: Defective ciliogenesis ↓ Autophagy	**Rapamycin**, 10 mg/kg daily, starting after seizure onset for 2 weeks: rescued ciliogenesis	Park et al., 2018
*mTOR L2427P (GOF)	E14	E18, P7: MP21: C	P21, L2/3 PN: ↓ Spine density	>P21: Spontaneous seizures	E18: Translational dysregulation	**Rapamycin**, 5 mg/kg daily, E14-E17: rescued M**eIF4E shRNA KD:** rescued M, C, spine density; ↓ seizures **Metformin (eIF4E inhibitor)**, 200 mg/kg daily, P14-56**:** rescued C; ↓ seizures **Metformin**, 200 mg/kg daily, P84-114**:** ↓ seizures **ADK shRNA KD** or **5-ITU (ADK inhibitor)**, 1 or 2.6 mg/kg, 2x daily for 10 days**:** ↓ seizures	Kim et al., 2019
*mTOR A1459D (GOF)	E14.5	E18.5: M, C	–	–	–	–	Hanai et al., 2017
*mTOR C1483Y (GOF)	E14	E16, E18: M>P56: C	–	>P21: Spontaneous seizures	>P56: Defective ciliogenesis ↓ Autophagy	**Rapamycin**, 10 mg/kg daily,after seizure onset for 2 weeks: rescued C, ciliogenesis defects **Ofd1 shRNA KD**: rescued M, ciliogenesis defects; no rescue of C, seizures **Wnt5a expression**: rescued M	Park et al., 2018
*mTOR C1483Y (GOF)	E14	E18, P7: MP21: C	P21, L2/3 PN: ↓ Spine density	>P21: Spontaneous seizures	E18: Translational dysregulation	**Rapamycin**, 5 mg/kg daily, E14-E17: rescued M**eIF4E shRNA KD:** rescued M, C, spine density; ↓ seizures **Metformin (eIF4E inhibitor)**, 200 mg/kg daily, P14-56**:** rescued C; ↓ seizures **Metformin**, 200 mg/kg daily, P84-114**:** ↓ seizures **ADK shRNA KD** or **5-ITU (ADK inhibitor)**, 1 or 2.6 mg/kg, 2x daily for 10 days**:** ↓ seizures	Kim et al., 2019
*mTOR C1483Y (GOF)	E13.5, SSC	E17.5: M, C	–	–	–	–	Tarkowski et al., 2019
*mTOR L1460P (GOF)	E13.5, SSC	E17.5: M, C	–	–	–	–	Tarkowski et al., 2019
*mTOR S2215Y (GOF)	E13.5, SSC	E17.5: M, C	–	–	–	–	Tarkowski et al., 2019
*mTOR R2505P* (GOF)	E13.5, SSC	E17.5: M, C	–	–	–	–	Tarkowski et al., 2019
*mTOR L2427T* (GOF)	E13.5, SSC	E17.5: M	–	–	–	–	Tarkowski et al., 2019
*mTOR S2215F (GOF)	E15 (rat)	E20: MP28: C	–	–	–	–	Pelorosso et al., 2019
*Stradα* (shRNA KD)	E14	E17, E19: M	–	–	–	–	Orlova et al., 2010
*Stradα* (shRNA KD)	E14	E19: M	–	–	–	**Rapamycin**, 5mg/kg daily, E15-E19: rescued M	Parker et al., 2013
*Depdc5*(CRISPR/Cas9 KO)	E14.5	E18.5: MP21-P63: M, CP20-P24: D	P20-P24, L2/3 PN:No change-Spine density ↑ Spine head width No change-sEPSC frequency ↑ sEPSC amplitude ↑ Capacitance ↓ R_input_ ↓ I/O (= ↑ rheobase)	>P21: Spontaneous seizures	–	**Rapamycin**, 1 mg/kg single injection at E15: rescued M	Ribierre et al., 2018
*Depdc5*(CRISPR/Cas9 KO)	E13-E14 (rat)	P21-30: C	P21-28, L2/3 PN: No change-RMP ↓ R_input_ Doublet AP firing	>P60: Spontaneous seizures	–	**Everolimus**, P10-21: rescued C	Hu et al., 2018
*Depdc5*^floxed/mutant^,Cre IUE, 2-hit model	E14.5	P15: CP42: M, C, D	–	P42:↓ Seizure threshold	–	***Depdc5 WT*** **or** ***Depdc5 Q542P (*****GOF*****)*** **expression:** rescued C ***Depdc5 F164 del*** **(LOF) expression:** no rescue of C	Dawson et al., 2020
*Nprl3*(CRISPR/Cas9 KO)	E14	P3: M, C>P35: C	–	>P35:↑ Cortical excitability↓ Seizure threshold	–	**Rapamycin**, 1 mg/kg single injection at E15: rescued M, C	Iffland et al., 2020

The table is organized by position of genes in the PI3K-mTOR pathway and GATOR1 complex, variant, and date of publication. All gene variants activate mTORC1 signaling. Stars in front of gene name denote human de novo somatic (^*^) or germline (^**^) mutations that have been identified in MCD (e.g., HME, FCDII) and epilepsy. All studies were done in mice unless noted otherwise. Targeted cortical areas are listed if they were specified in the original publication. Ages refers to when IUE was performed and the timepoints of pathological and/or behavioral evaluation. GOF, gain-of-function; LOF, loss-of-function; WT, wildtype; KO, knockout; KD, knockdown; E, embryonic day; P, postnatal day; SSC, somatosensory cortex; CC, cingulate cortex; mPFC, medial prefrontal cortex; L2/3 PN, layer 2/3 pyramidal neurons; RMP, resting membrane potential; R_input_, input resistance; R_membrane_, membrane resistance; I/O, input/output; mIPSC, miniature inhibitory postsynaptic current; mEPSC, miniature excitatory postsynaptic current; sEPSC, spontaneous excitatory postsynaptic current; eEPSC, evoked excitatory postsynaptic current; AP, action potential; DN, dominant negative; NF, non-functional.

The authors apologize for this error and state that this does not change the scientific conclusions of the article in any way. The original article has been updated.
